# Encouraging cartilage production

**DOI:** 10.7554/eLife.57239

**Published:** 2020-05-06

**Authors:** H Scott Stadler

**Affiliations:** 1 Orthopaedics and Rehabilitation, Oregon Health Science UniversityPortlandUnited States; 2 Skeletal Biology Research Center, Shriners Hospital for ChildrenPortlandUnited States

**Keywords:** mesenchymal stem cells, tissue engineering, regenerative medicine, rnf144a-as1, stem cells, Human

## Abstract

A long non-coding RNA called *GRASLND* is essential to help stem cells create stable cartilage.

**Related research article** Huynh NPT, Gloss CC, Lorentz J, Tang R, Brunger JM, McAlinden A, Zhang B, Guilak F. 2020. Long non-coding RNA *GRASLND* enhances chondrogenesis via suppression of interferon type II signaling pathway. *eLife* **9**:e49558. doi: 10.7554/eLife.49558

Recently watching a rerun of the 2016 Olympics gymnastics finals, I could not help marveling at the way the joints of the athletes could withstand so many gravity-defying leaps, twists, and landings. These feats are possible because the ends of our bones are covered by articular cartilage, a smooth tissue that allows fluid, pain-free movement. This tissue is made by specialized cells secreting proteins that trap water and form an extracellular matrix which cushions joints.

When articular cartilage wears away, for example in degenerative diseases such as osteoarthritis, movements become painful and quality of life drops severely. Yet, these conditions are increasingly common – in the United States alone, it is predicted that more than 78 million people could be affected by 2040 (Hootman et al., 2016).

Cartilage is not connected to the nervous system or to blood and lymphatic vessels, which means the tissue heals poorly when damaged. Most therapies for osteoarthritis therefore work by preserving the remaining cartilage or preventing further loss. Once the cartilage is lost, few interventions exist: surgeons can carefully damage the bone to promote the creation of new tissue, they can graft bone and cartilage obtained from a donor, or they can completely replace the joints with artificial ones (Steadman et al., 2001; Toh et al., 2014; Bugbee et al., 2016; Migliorini et al., 2020). However, these interventions may not be durable, and they are limited by factors such as the availability of donor tissue and the age or health condition of the patient.

Another, lab-based approach is to harvest mesenchymal stem cells or chondroprogenitor cells from patients, and then 'coax' these to create cartilage that can be implanted in the individual (Migliorini et al., 2020). However, one challenge associated with this method is the stability of the resulting cartilage: over time, it can change into bone, reducing the function of the repaired joint.

Long non-coding RNAs are molecules that regulate an array of genetic events in the cell, and it was reported recently that these sequences are essential to keep cartilage stable: for instance, several long non-coding RNAs are activated in mesenchymal stem cells that produce cartilage (Barter et al., 2017; Huynh et al., 2019). Now in eLife, Farshid Guilak and colleagues – including Nguyen Hyunh as first author – report having identified a long non-coding RNA called *GRASLND* which encourages mesenchymal stem cells to produce molecules that form cartilage (Huynh et al., 2020).

First, the team (which is based at Washington University in St. Louis, the St. Louis Shriners Hospital, Duke University and Vanderbilt University) designed RNA molecules that were used to deactivate *GRASLND* in mesenchymal stem cells. As a result, the production of cartilage decreased and these cells started to show a molecular profile associated with bone formation. These results demonstrate that, in these cells, *GRASLND* is required to maintain a cartilage-forming program (Figure 1).

**Figure 1.**
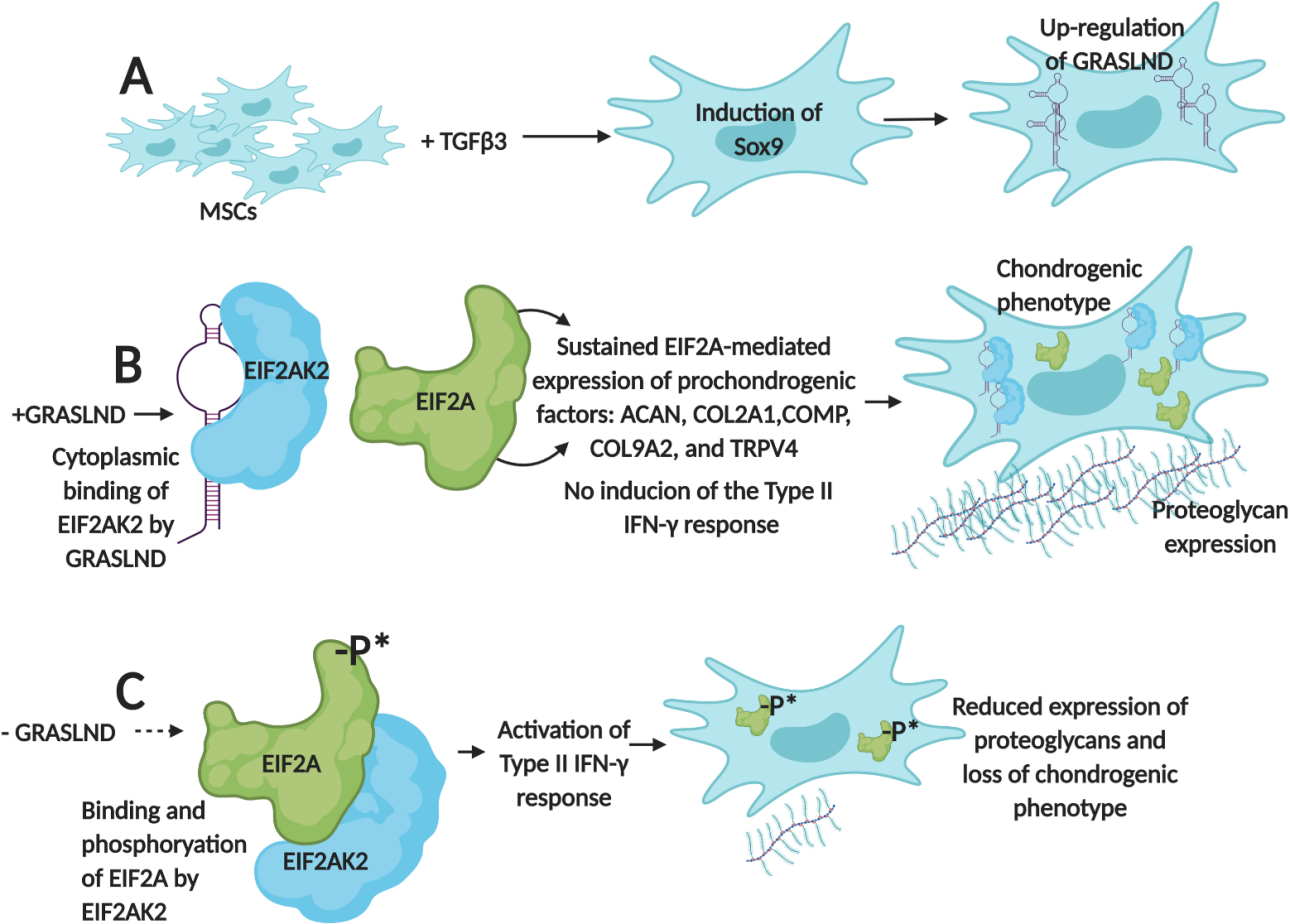
*GRASLND* helps mesenchymal stem cells to create cartilage by suppressing IFN-signaling. (**A**) Exposing mesenchymal stem cells (MSCs) to the growth factor TGFβ3 activates the expression of the *Sox9* gene, which triggers the production of a long non-coding RNA called *GRASLND.* (**B**) *GRASLND* binds to the kinase EIF2AK2 (blue), which blocks the inhibitory phosphorylation of the protein EIF2A (green). This, in turn, promotes the expression of 'prochondrogenic factors' that encourage the production of molecules, such as proteoglycans, which form cartilage; the cell is said to have a 'chondrogenic' phenotype. (**C**) When *GRASLND* is depleted from mesenchymal stem cells, the kinase EIF2AK2 probably phosphorylates EIF2A (represented here by the ‘-P*’). This activates the Type II IFN-γ response, which ultimately leads to a reduction in proteoglycan expression and a loss of the chondrogenic phenotype. *GRASLND*: glycosaminoglycan regulatory associated long non-coding RNA; EIF2A: eukaryotic translation initiation factor two alpha; EIF2AK2: EIF2A kinase; TGFβ3: transforming growth factor beta 3. Figure created using BioRender (BioRender.com).

Further experiments showed that *GRASLND* interacts with EIF2AK2, a kinase that normally inhibits a protein known as EIF2A, which triggers a molecular cascade called the type II IFN-γ signaling pathway (Samuel, 1979; Platanias, 2005). This pathway is essential for the immune system, but some of its elements, such as a cytokine called IFN-γ, also help to stimulate bone formation (Duque et al., 2011).

When *GRASLND* binds EIF2AK2, it probably stops this kinase from acting on EIF2A; this suppresses IFN activity while allowing the genes that promote the production of cartilage to be expressed (Figure 1B). On the other hand, Hyunh et al. find that removing *GRASLND* is associated with an increase in the expression of genes under the control of IFN-γ (Figure 1C). As IFN-γ promotes bone formation, these findings explain why depleting mesenchymal stem cells of *GRASLND* leads to more bone production.

Finally, Hyunh et al. used data mining to show that, in diseased cartilage, genes regulated by IFN are expressed more abundantly. This suggests that IFN-signaling may be directly responsible for the production of the abnormal, bony nodules that are often present in osteoarthritic cartilage.

Overall, these results indicate that — at least in vitro — *GRASLND* is an important modulator of type II IFN-γ signaling that is necessary for cartilage differentiation. They also highlight that this pathway may be involved in diseases of the cartilage. If so, the interaction between *GRASLND* and EIF2AK2 could be an important pharmacological target. Exploring this possibility will first require comparing the expression of *GRASLND* in healthy and diseased cartilage.

*GRASLND* has only been found in primates, but related long non-coding RNAs could be identified in other species by spotting the motifs that *GRASLND* needs to interact with EIF2AK2. In turn, this knowledge could pave the way for better animal models to study how this class of long non-coding RNAs is involved in degenerative joint diseases.

## References

[bib1] Barter MJ, Gomez R, Hyatt S, Cheung K, Skelton AJ, Xu Y, Clark IM, Young DA (2017). The long non-coding RNA *ROCR* contributes to SOX9 expression and chondrogenic differentiation of human mesenchymal stem cells. Development.

[bib2] Bugbee WD, Pallante-Kichura AL, Görtz S, Amiel D, Sah R (2016). Osteochondral allograft transplantation in cartilage repair: graft storage paradigm, translational models, and clinical applications. Journal of Orthopaedic Research.

[bib3] Duque G, Huang DC, Dion N, Macoritto M, Rivas D, Li W, Yang XF, Li J, Lian J, Marino FT, Barralet J, Lascau V, Deschênes C, Ste-Marie LG, Kremer R (2011). Interferon-γ plays a role in bone formation in vivo and rescues osteoporosis in ovariectomized mice. Journal of Bone and Mineral Research.

[bib4] Hootman JM, Helmick CG, Barbour KE, Theis KA, Boring MA (2016). Updated projected prevalence of self-reported doctor-diagnosed arthritis and arthritis-attributable activity limitation among US adults, 2015-2040. Arthritis & Rheumatology.

[bib5] Huynh NPT, Zhang B, Guilak F (2019). High‐depth transcriptomic profiling reveals the temporal gene signature of human mesenchymal stem cells during chondrogenesis. The FASEB Journal.

[bib6] Huynh NPT, Gloss CC, Lorentz J, Tang R, Brunger JM, McAlinden A, Zhang B, Guilak F (2020). Long non-coding RNA *GRASLND* enhances chondrogenesis via suppression of interferon type II signaling pathway. eLife.

[bib7] Migliorini F, Berton A, Salvatore G, Candela V, Khan W, Longo UG, Denaro V (2020). Autologous chondrocyte implantation and mesenchymal stem cells for the treatments of chondral defects of the knee - A systematic review. Current Stem Cell Research & Therapy.

[bib8] Platanias LC (2005). Mechanisms of type-I- and type-II-interferon-mediated signalling. Nature Reviews Immunology.

[bib9] Samuel CE (1979). Mechanism of interferon action: phosphorylation of protein synthesis initiation factor eIF-2 in interferon-treated human cells by a ribosome-associated kinase processing site specificity similar to hemin-regulated rabbit reticulocyte kinase. PNAS.

[bib10] Steadman JR, Rodkey WG, Rodrigo JJ (2001). Microfracture: surgical technique and rehabilitation to treat chondral defects. Clinical Orthopaedics and Related Research.

[bib11] Toh WS, Foldager CB, Pei M, Hui JHP (2014). Advances in mesenchymal stem cell-based strategies for cartilage repair and regeneration. Stem Cell Reviews and Reports.

